# The effect of morpheme positional frequency on tibetan novel word acquisition: An eye-tracking study

**DOI:** 10.1371/journal.pone.0337615

**Published:** 2025-11-26

**Authors:** Dingyi Niu, Jing Tian

**Affiliations:** 1 School of Education, Xizang University, Lhasa, China; 2 School of Educational Science, Shanxi Normal University, Taiyuan, China; Father Muller Charitable Institutions, INDIA

## Abstract

Word segmentation is crucial for reading in unspaced languages like Tibetan, where readers rely on high-level cues like morpheme positional frequency (the statistical likelihood of a morpheme appearing at the beginning or end of a word). Using a novel word learning paradigm, this study investigated whether initial and final morpheme positional frequency facilitate word segmentation and lexical recognition in Tibetan. In two eye-tracking experiments, participants read sentences containing novel words manipulated for initial (Experiment 1) or final (Experiment 2) morpheme positional frequency across learning and testing phases. Results showed that while initial morpheme positional frequency influenced saccade target selection and the late stage of lexical recognition, final morpheme positional frequency primarily affected late stage of lexical recognition. Reading phase improved efficiency across all measures. The effects of morpheme positional frequency and the reading phase were independent at both early and late stages of lexical recognition. Findings provided evidence that morpheme positional frequency may serve as a functional word segmentation cue in the context of Tibetan novel word acquisition.

## Introduction

### 1. Definition and importance of word segmentation

Word segmentation is the process of breaking down continuous text into independent lexical units, which serves as a cognitive process essential for efficient reading comprehension and lexical processing across different writing systems [1,2]. For languages with inter-word spaces, such as English and Spanish, readers rely on these spaces as word boundary markers [3]. In contrast, for scripts like Tibetan and Chinese that lack inter-word spaces, there are no obvious visual boundary markers between words [4]. Consequently, readers must depend on other cues, such as morpheme position, word frequency, and lexical expectation, to perform word segmentation [5]. Word segmentation plays a crucial role in the reading process; it not only affects lexical recognition and reading fluency but also has a significant impact on the acquisition of novel words and the development of reading skills [6]. For example, research has demonstrated that inserting inter-word spaces in Chinese text facilitates both children’s and adults’ learning of novel pseudowords within sentence contexts, with benefits maintained over subsequent encounters, particularly for child readers [7,8].

The importance of word segmentation in reading is reflected in several aspects: 1. Promoting reading efficiency: Studies have shown that reading speed under conditions with inter-word spaces is significantly faster than under conditions with no spaces or non-word spaces [9]. This indicates that word segmentation enables readers to perform lexical recognition and sentence comprehension more efficiently. 2. Aiding lexical recognition: In Chinese reading, the absence of inter-word spaces requires readers to rely on word segmentation to identify words. Using eye-tracking methodology, word segmentation has been shown to not only facilitate the early recognition of words but also reduces the number of regressions, thereby improving reading fluency [10]. 3. Supporting cognitive processing: Word segmentation is a vital cognitive process in Chinese reading, involving the combined effects of factors like morpheme positional frequency and word frequency. These cues help readers process information effectively during reading [11]. 4. Affecting reading comprehension: Word segmentation influences not only reading speed but also reading comprehension significantly. A study examined Chinese preschool children (5–6 years old) reading Chinese sentences under both spaced and unspaced conditions using eye-tracking technology, and showed that word segmentation can optimize the early reading comprehension scores of children in large classes [12]. 5. Role in language learning: For second-language learners, word segmentation plays an important role in reading. Studies have indicated that word segmentation promotes the Chinese reading speed and comprehension ability of American international students [13,14]. 6. Regulating eye movement behavior: Word segmentation has a significant effect on eye movement behavior. For example, the average fixation duration under inter-word space conditions is significantly shorter than under normal conditions, indicating that word segmentation can reduce the number of regressions and improve reading efficiency [15].

### 2. Classification of word segmentation cues

Word segmentation cues include low-level word segmentation cues and high-level word segmentation cues. This distinction follows theoretical frameworks proposed in the literature on reading across different writing systems [16,17], where low-level cues refer to perceptually-driven visual markers, while high-level cues involve linguistically-driven statistical and contextual information [18].

#### 2.1. Low-level word segmentation cues.

Low-level word segmentation cues are perceptually salient, visually-based markers that provide explicit spatial demarcation of word boundaries in text [19]. These cues operate primarily at the level of orthographic processing, preceding lexical access, and function independently of linguistic context or semantic information [20,21]. In alphabetic scripts, the inter-word space is a typical low-level visual word segmentation cue. It explicitly marks the boundaries between words, helping readers to perform word segmentation quickly [15].

Low-level cues primarily assist word segmentation through directly visible visual boundary markers in the text, using physical separation. Research across multiple writing systems has demonstrated the effectiveness of various low-level segmentation markers, including inter-word spacing in alphabetic languages [3,22], visual highlighting and color alternation in unspaced scripts [23,24], and other spatial modifications [25,26]. They manifest in the following forms: 1. Inter-word space markers: This is the most direct form of visual separation. When spaces are inserted into Chinese text to mark word boundaries (e.g., “中文 阅读” [Chinese reading]), research has found that inter-word spaces significantly enhance the reading efficiency of beginners, such as primary school students. The mechanism is that they provide clear visual boundaries for lexical recognition [5,15]. Experimental evidence showed that Chinese third-grade students found reading Chinese text with inter-word spaces to be of comparable difficulty to reading normal, unspaced text. However, non-word spaces (e.g., “中 文阅 读” [C-hinese rea-ding]) severely interfered with students who had lower reading skills [15]. 2. Alternating color markers: Color-based boundary demarcation (e.g., alternating red and green) avoids the changes in text spatial distribution caused by spaces. Studies found that word-appropriate color marking maintains reading efficiency comparable to normal text; however, non-word color marking interfered with reading [27,28]. Critically, this color marking effect only affects the visual information processing stage and is not influenced by word frequency, which suggests its role is confined to the early stage of lexical recognition [27]. This pattern has been replicated in Tibetan reading, where color alternation markings facilitated lexical recognition but did not affect saccade target selection [28]. 3. Gray bar markers: Using gray bars to replace spaces for marking boundaries maintains the spatial distribution of the sentence. Experiments showed that their effect is similar to that of inter-word spaces but avoids the interference on reading caused by changes in physical space [15].

#### 2.2. High-level word segmentation cues.

Unlike low-level word segmentation cues such as inter-word spaces, high-level word segmentation cues refer to the grammatical and semantic information that readers rely on to assist in word segmentation during the reading process. According to theoretical frameworks of word segmentation in unspaced languages [17,29], high-level cues operate through interactive activation processes involving both bottom-up character-level processing and top-down lexical and contextual information [30]. Li et al.‘s (2009) computational model of Chinese word segmentation proposes that word identification and segmentation occur simultaneously through competition among candidate words activated by character-level input, modulated by lexical frequency and contextual predictability [1]. This interactive process contrasts with the serial word identification assumed in models of alphabetic reading like E-Z Reader [31,32].

For instance, when we see the string of unspaced English characters—Thesunriseseverymorning—we can still understand the meaning of the sentence, albeit with some difficulty: The sun rises every morning. This is because we search for familiar character combinations within this string and attempt to segment it into meaningful and plausible words to comprehend its meaning. In this process, we are using high-level word segmentation cues. These cues can help readers correctly divide words in a sentence by leveraging semantic understanding, grammatical structure, and word familiarity, especially when faced with ambiguity or a lack of clear word boundaries [33]. Empirical evidence from Chinese reading demonstrates that readers utilize statistical regularities, morpheme positional probabilities, and lexical context to segment words during natural reading [18,34]. Similarly, research in Tibetan reading suggests that, despite the absence of inter-word spacing, readers rely on linguistic cues to achieve efficient word segmentation [4,35]. For languages like Tibetan and Chinese, which naturally lack low-level word segmentation cues, high-level cues are particularly important.

Among high-level word segmentation cues, morpheme positional frequency is the most extensively studied. When we read a stretch of unspaced English text, we are highly likely to treat “pre-” as the beginning of a word and “-ing” as the end of a word. This is because “pre-” commonly appears at the beginning of words, while “-ing” commonly appears at the end. This process of judging word boundaries based on whether a morpheme more frequently appears at the beginning or end of a word is word segmentation through morpheme positional frequency. In languages with a finite number of morphemes, such as Chinese and Tibetan, morpheme positional frequency is defined as the statistical frequency of a Chinese character (or a Tibetan morpheme) appearing in a specific position (initial or final) within a two-character word (or two-morpheme word). In Chinese, the calculation method for morpheme positional frequency is: the number of two-character words where a specific character appears in the initial position, divided by the total number of two-character words that the character helps to form [11]. For example, the character “精” (jīng, essence) predominantly appears at the beginning of two-character words, yielding a high initial positional frequency (>0.85), as in words like “精神” (jīngshén, spirit) [11].

Characters with high positional frequency (like “精”, which often appears in the initial position) provide readers with implicit word boundary cues, helping them to segment words quickly [11,36]. Experimental studies have shown that when the initial and final morpheme positional frequency of a novel word are consistent with its actual position (e.g., the initial morpheme often appears at the beginning of words, and the final morpheme often appears at the end), lexical recognition is fastest [5,37]. Conversely, inconsistent positional frequency (e.g., an initial morpheme that often appears at the end of words) significantly prolongs fixation duration [37]. This finding revealed the cognitive mechanism of morpheme positional frequency as an internal statistical cue: through long-term language exposure, readers internalize the positional distribution patterns of characters within words and automatically activate this statistical knowledge during lexical recognition, thereby facilitating the rapid identification of word boundaries.

### 3. Word segmentation cues in different languages

#### 3.1. The role of low-level word segmentation cues in word segmentation.

**3.1.1. The Role of Spaces in Word Segmentation:** When reading alphabetic scripts like English and Spanish, readers heavily rely on the inter-word space as a low-level word segmentation cue [38,39]. This reliance on spacing is observed broadly across alphabetic writing systems that employ inter-word spaces, including not only Latin-based languages (English, Spanish, German) but also Greek, Cyrillic-based languages (Russian), and Hebrew [40,41]. In these languages with inter-word spaces, spaces have three core functions: providing word boundary cues [39], which reduces lexical ambiguity and facilitates parallel word identification in parafoveal vision [42]; guiding eye movement landing positions [38], enabling readers to target optimal viewing positions within words and plan efficient saccades [43]; and reducing letter visual crowding [44], thereby improving letter recognition accuracy through lateral interference reduction [45].

When spaces are removed, the reading speed in both languages decreases significantly, by 40−70% [46], and this leads to disordered eye movement patterns [38]. Sheridan et al. (2016) conducted a study on English, comparing eye movement trajectories for normal spaced text and unspaced text. The study recruited native English-speaking university students (n = 24) as participants and they found that removing spaces led to: a decrease in reading speed, as the amount of information processed per unit of time was reduced (average decrease of 20%−30%) [47]; prolonged fixation durations, with single fixation durations increasing by 15%−25%, reflecting increased difficulty in lexical recognition [47]; abnormal skipping patterns, with the word skipping rate decreasing by 30%, indicating that parafoveal processing was hindered [47]; and shortened saccade distances by 10%−15%, with the initial fixation position being closer to the beginning of the word (a shift of 0.3-0.5 letter positions) [47]. In unspaced text, the word frequency effect appeared later (emerging at 60 ms in normal text, but delayed to 100 ms in unspaced text), which showed that lexical recognition speed was slower [47].

Similar to the findings of Sheridan et al., an experiment by Juhasz et al. (2008) found that in English reading, the absence of spaces increased the first fixation duration on short words (≤4 letters) by 22% [39]. This particularly strong effect for short words occurs because shorter words have fewer internal orthographic features to distinguish them from adjacent words, making them more dependent on external boundary cues (spaces) for rapid identification [48]. Additionally, short words typically receive less parafoveal preview benefit, making immediate visual segmentation cues more critical [49]. This further demonstrated the primary role of spaces for word segmentation in English reading.

Research in Spanish yielded similar results to those in English, demonstrating broader generalizability. Toshima et al. (2011) used Spanish as the research language, asking native Spanish-speaking participants to read unspaced Spanish sentences. The results from multiple-choice comprehension questions administered after each sentence showed: a 35% increase in word segmentation errors and a decrease in comprehension accuracy, with the correctness rate for reading comprehension questions on unspaced text dropping by 18% [50]. The absence of spaces forced readers to allocate more cognitive resources to word segmentation, which crowded out resources for semantic integration [50]. This indicates that in languages with inter-word spaces, the space is a critical word segmentation cue that is vital for reading comprehension.

**3.1.2. The Role of the Saliency of Kanji Characters in Word Segmentation:** For scripts that do not have inter-word spaces, other low-level word segmentation cues may exist. For instance, in Japanese text, the visual contrast between Kanji (ideographic characters) and Kana (syllabic scripts) serves as a salient boundary marker [51,52]. Early research by Kajii, Nazir, and Osaka (2001) demonstrated that Kanji characters, due to their greater visual complexity and morphographic nature, function as visual anchors that guide eye movements and facilitate word boundary identification in mixed-script Japanese text [52]. The boundaries between Hiragana, Katakana, and Kanji can serve as a basis for judging word boundaries.

Fujii and Ishikawa (2001) found that the word segmentation accuracy for combinations like “CK” (Kanji + Katakana) and “CA” (Kanji + Alphabet) was higher, reaching 95% in a Japanese-English cross-language information retrieval task where native Japanese speakers (n = 30) segmented compound technical terms [53]. For instance, in the character string “東京タワー” (Tokyo Tower), it is easier for us to identify the word boundary between “東京” and “タワー” [53]. This is because Kanji characters have a higher visual complexity compared to Hiragana and Katakana [54], making them easy to form “visual anchors” in the text. An eye movement experiment [14] showed that native Japanese speakers did not need space assistance in mixed Kanji-Kana text because Kanji provides word boundary cues; however, inserting spaces into purely Kana text could improve reading efficiency by 26% (measured as a 26% reduction in total sentence reading time). This indirectly suggests that Japanese readers rely on the saliency of Kanji for word segmentation.

#### 3.2. High-level word segmentation cues: The role of morpheme positional frequency in word segmentation.

Morpheme positional frequency is a high-level word segmentation cue (e.g., morpheme positional frequency), often found in languages without low-level cues (e.g., spaces between words), such as Chinese, Tibetan, Thai, and the Yi script. Research on the effect of morpheme positional frequency on word segmentation has so far been concentrated on Chinese.

**3.2.1. The Effect of Morpheme Positional Frequency in Natural Reading:** First, morpheme positional frequency (the statistical frequency of a character appearing in a specific position) affects word segmentation during natural reading. Liang Feifei et al. conducted two experiments with 48 adults and 48 third-grade children who were native speakers of Chinese as participants. In Experiment 1, they selected two target words with similar meanings to control for semantic processing difficulty and contextual predictability, ensuring that any observed differences in reading times would be attributable to positional frequency manipulation rather than semantic factors [55]. They then manipulated the frequency of the initial character’s appearance. For example, in “湖水” (húshuǐ, lake water) and “泉水” (quánshuǐ, spring water), the character “湖” (hú) has a higher frequency of appearing at the beginning of a word, while “泉” (quán) has a lower frequency. The target word was then embedded in the same sentence, and the participants’ eye movements were observed as they read the two sets of sentences. The results showed that the initial morpheme positional frequency did not have a significant effect on word segmentation or lexical recognition. In Experiment 2, they manipulated the second character of the target word. The results revealed that the final morpheme positional frequency of the target word had an effect on the early stage of lexical recognition in adult reading, meaning it could help readers successfully complete word segmentation and lexical recognition. In children’s reading, however, the effect of this frequency appeared in the later stage. This may reflect differences in reading proficiency and cognitive processing between adults and children. Experiment 1 and Experiment 2 confirmed that the positional frequency of the final morpheme (but not the initial morpheme) has a significant impact on word segmentation and lexical recognition [55].

**3.2.2. The Effect of Morpheme Positional Frequency in the Acquisition of Novel Words:** Second, morpheme positional frequency affects word segmentation during the acquisition of novel words. Research in this area has commonly used the novel word learning paradigm. In this paradigm, novel words are created according to certain rules, and the positional frequency of their constituent parts (i.e., characters) is manipulated, including the initial morpheme positional frequency and final morpheme positional frequency of the novel word. The newly created novel words are then embedded in sentences, and participants are asked to read and learn them. Eye movement measures—including fixation duration, gaze duration, and total reading time—are considered reliable indicators of word segmentation processes because they provide millisecond-by-millisecond temporal indices of ongoing cognitive processing during natural reading [56,57]. Specifically, longer fixation durations and increased refixations typically reflect difficulty in word segmentation and lexical identification, while shorter fixations indicate more efficient boundary detection and lexical access [40,58]. These measures have been extensively validated as sensitive indices of lexical processing difficulty and word boundary ambiguity [59,60]. By observing participants’ eye movements during this process, one can infer the potential influence of morpheme positional frequency on word segmentation in the acquisition of novel words [61].

Liang Feifei conducted a study with 15 university students who were native Chinese speakers and constructed three types of novel words based on the positional frequency of each character. In the first type of novel word, the position of the constituent characters was consistent with their positional frequency; in the second type, the position was inconsistent; in the third type, the frequency of the constituent characters appearing in the initial position was similar to their frequency of appearing in the final position. Each novel word was embedded in a sentence. The experiment included two phases: a learning phase and a testing phase. In the learning phase, participants were only required to read the sentences containing the novel words. In the testing phase, participants were not only required to read the sentences but also to complete a semantic categorization task, such as determining whether the novel word belonged to the category of animals, plants, furniture, or another given category. The learning outcomes of the participants could be evaluated through this task. During the learning and testing phases, participants’ eye movement behaviors were recorded using an eye-tracker. Based on an analysis of skipping rate, fixation duration, fixation count, and fixation position, Liang Feifei concluded that in Chinese reading, morpheme positional frequency is an effective linguistic word segmentation marker. Its effect spans the entire process of lexical recognition, with a broad scope and long duration of action [10].

**3.2.3. The Effect of Morpheme Positional Frequency in Overlapping Ambiguous Strings:** Furthermore, morpheme positional frequency affects word segmentation in overlapping ambiguous strings. Cao Haibo et al. manipulated the initial and final morpheme positional frequency of overlapping ambiguous strings (e.g., “邮差距” yóu-chā-jù, which can be segmented as “邮差” yóu-chāi, postman, or “差距” chā-jù, gap) and asked participants to quickly report the pronunciation of the middle character. The results showed that readers tended to report the pronunciation of the character on the side with the higher morpheme positional frequency. In another of their experiments, they manipulated the initial and final morpheme positional frequency of overlapping ambiguous strings (e.g., “惹祸害” rě-huò-hài, which can be segmented as “惹祸” rě-huò, to cause trouble, or “祸害” huò-hài, to harm) and asked participants to report one word from the ambiguous string. The results found that readers tended to report the word on the side with the higher morpheme positional frequency. The results of both experiments indicate that morpheme positional frequency is an important linguistic word segmentation cue for Chinese readers. Readers can use the initial and final morpheme positional frequency to segment overlapping ambiguous strings, and both cues play a role in the process of lexical recognition [62].

**3.2.4. The Effect of Morpheme Positional Frequency in Reading Aloud:** Finally, morpheme positional frequency also influences word segmentation during reading aloud. Lian Kunyu et al., through two experiments, manipulated the level (high or low) of initial and final morpheme positional frequency to investigate whether Chinese readers use this information for word segmentation during reading aloud. The results found that under reading-aloud conditions, participants could utilize initial morpheme positional frequency to perform word segmentation [63].

### The effect of morpheme positional frequency in other languages

For Japanese and Thai, two languages without inter-word spaces, there have also been findings of morpheme positional frequency influencing word segmentation. Murata et al. found that in Japanese reading, certain morphemes may appear frequently at the beginning or end of words, and these morphemes can provide readers with word boundary information [64]. Higashiyama et al. suggested that morpheme positional frequency, similar to spaces, could potentially serve as a cue for word segmentation. In the absence of explicit inter-word spaces, readers would rely on morpheme positional frequency to assist in word segmentation [65]. Similar findings exist in Thai reading. Kasisopa et al. (2013) found that in Thai reading, if a high-frequency character appears at the beginning or end of a word, readers’ saccade landing positions would be closer to the word center, optimizing word segmentation efficiency [66]. However, research on this topic in Japanese and Thai is not as extensive as in the field of Chinese.

Besides Japanese and Thai, there are many other languages without inter-word spaces, such as Tibetan and the Yi script [4,67]. These languages may all possess non-space word segmentation cues.

### 4. Tibetan word segmentation cues

Tibetan is a phonetic script belonging to the Sino-Tibetan language family. The Tibetan writing system consists of 30 basic consonant letters and 4 basic vowel symbols, which can be combined in various ways to represent different morphemes. In Tibetan, the morpheme “བོད” (meaning “Tibet” or “Tibetan”) is formed by the combination of the consonant “བ” with the vowel symbol “ོ” to create “བོ”, which is then combined with the consonant “ད”. Tibetan has its own unique morpheme segmentation cue, which is the tsheg (“་”). Tshegs are used consistently to mark morpheme boundaries within multi-morpheme words [68]. For example, in the Tibetan language, the word “བོད་ཡིག” (meaning “Tibetan language”) is composed of “བོད” and “ཡིག” (meaning “writing” or “script”). The tsheg between these two morphemes serves as a morpheme segmentation cue and also aids in lexical recognition [69]. Although these two morphemes together form a complete semantic unit, they must still be separated by a tsheg [4]. From the above introduction to Tibetan, it can be understood that while Tibetan has a cue for morpheme segmentation, it lacks a natural cue for word segmentation. Despite this absence of word boundary markers, this does not hinder native speakers from reading it [69].

Therefore, after discussing the effect of artificially inserted inter-word spaces as a low-level word-segmentation cue in Tibetan reading, Wang et al. speculated that Tibetan also possesses high-level word segmentation cues [4]. Wang et al. proposed that Tibetan, similar to Chinese, may also possess the high-level word segmentation cue of morpheme positional frequency [4].

### 5. Significance of the novel word learning paradigm for morpheme positional frequency

The novel word learning paradigm offers several unique advantages over other methods (such as natural reading tasks) when investigating the impact of morpheme positional frequency (i.e., the statistical frequency of a morpheme appearing at the beginning or end of a word) on word segmentation and lexical acquisition:

#### 5.1. Directly examining the initial stage of word segmentation and lexical acquisition.

The acquisition of novel words involves integrating entirely new vocabulary into a learner’s lexicon, a process highly dependent on word segmentation skills. Empirical studies in Chinese have demonstrated that morpheme positional frequency significantly affects novel word learning during natural reading [55,61]. Morpheme positional frequency, as an implicit cue, can help learners determine word boundaries, and the novel word learning paradigm provides a direct opportunity to observe the effect of this cue. In languages without explicit word boundaries, learners may rely on high-positional-frequency morphemes to infer the start and end positions of words, a process that is particularly evident in the acquisition of novel words [55,61].

#### 5.2. Controlling experimental variables to isolate the effect of morpheme positional frequency.

In natural reading tasks, the effect of morpheme positional frequency is often confounded with other factors (such as word frequency and contextual predictability), making it difficult to analyze its role in isolation. For example, in Chinese, the morpheme 湖 (hú, ‘lake’) has a high initial positional frequency, while the morpheme 泉 (quán, ‘spring’) has a low one. A simple comparison between existing words like 湖水 (húshuǐ, ‘lake water’) and 泉水 (quánshuǐ, ‘spring water’) is confounded by factors such as their differing word frequencies and orthographic complexity (i.e., stroke counts). To isolate the effect of positional frequency, a researcher could create two novel words, such as by pairing each morpheme with the same neutral character. By ensuring these two novel words are presented an equal number of times, any observed difference in processing can be more reliably attributed to the positional frequency of the initial morphemes (湖 vs. 泉). This controlled environment can effectively isolate the independent effect of morpheme positional frequency, providing clearer causal evidence [55,61].

#### 5.3. Real-time capture of cognitive processing.

The novel word learning paradigm, when combined with eye-tracking technology, can capture the cognitive dynamics of learners in real-time during word segmentation and lexical recognition. This paradigm often presents a novel word in different sentences six or even more times. By analyzing indicators such as skipping rates and fixation durations during the multiple exposures to the novel word, it is possible to precisely reveal how morpheme positional frequency affects the learning and processing of novel words. Such real-time data are more difficult to obtain in natural reading, as it rarely creates six similar reading processes [55,61].

In summary, using the novel word learning paradigm to study morpheme positional frequency in Tibetan reading will be particularly valuable. Given that Tibetan, like Chinese, lacks inter-word spacing, investigating whether Tibetan readers utilize morpheme positional frequency cues during novel word acquisition will be an efficient and robust method and could reveal fundamental similarities and differences in word segmentation mechanisms across unspaced writing systems.

### 6. Research significance

The theoretical significance of this study is mainly reflected in the following aspects: first, by systematically exploring the role of morpheme positional frequency in Tibetan, it is possible to verify the universality of this cognitive mechanism across different language systems, by comparing the findings with existing research on other languages, thereby enriching and refining the theoretical framework of word segmentation. Second, as a representative agglutinative language with a syllabic alphabet language of the Sino-Tibetan language family, an in-depth study of its word segmentation mechanism will address the current gap in empirical evidence and provide important empirical evidence for the cognitive processing mechanisms of Sino-Tibetan languages. Third, this study will advance the development of theories on cross-linguistic lexical acquisition, offering a new perspective for understanding the cognitive mechanisms of the acquisition of novel words in different language systems.

From a practical perspective, the results of this study will provide a scientific cognitive basis for Tibetan language teaching and learning, contributing to the optimization of Tibetan reading materials and the improvement of teaching methods. It will also offer methodological references for cognitive research on other minority languages, holding significant value for promoting the development of minority language education.

## Experiment 1

### Methods

#### Participants.

To determine an appropriate sample size, we conducted an a priori power analysis using GPower (Version 3.1.9.2) [70]. The study employed a 2 × 2 within-participants design, so we based our calculation on the “ANOVA: Repeated measures, within factors” model in F-tests. The parameters were set as follows: effect size f = 0.25 (medium effect), α = 0.05, statistical power (1-β) = 0.80, number of groups = 1, number of measurements = 4, and correlation among repeated measures = 0.5, assuming sphericity (nonsphericity correction ε = 1). The calculation showed that the minimum sample size required to achieve the desired power was 24. It should be noted that the subsequent analysis used the lme4 package in R to conduct a 2 × 2 linear mixed model (LMM) analysis of fixed effects [71]. Although this analysis is more suitable for within-participants designs, LMM was chosen over traditional ANOVA as it offers greater flexibility, particularly in its ability to simultaneously account for both participant and item variability through random effects and to handle missing data points without excluding entire participants [71]. Therefore, the sample size calculated by GPower might have a slight margin of error. Consequently, we ultimately recruited 60 participants. After excluding samples (n = 4) with an eye-tracking data collection rate below 85%, the number of valid participants was 56. These 56 native Tibetan speakers (28 male, 28 female, all of whom were also proficient in Mandarin Chinese) were from Tibetan communities in Shanxi Province, China, with an age range of 18–55 years (M = 34.7, SD = 9.1). All participants were right-handed (a criterion to minimize variability in brain lateralization for language processing), had normal or corrected-to-normal vision, and had no history of neurological disorders. All participants signed an informed consent form before the experiment and received 50 RMB as compensation after the experiment.

#### Ethics statement.

This prospective study was approved by the university-level Science and Technology Ethics Committee, Shanxi Normal University (Approval No. 2025−0701, Date: July 20, 2025). Participant recruitment started on 20 July 2025 and ended on 30 July 2025, and all study procedures were conducted at Shanxi Normal University. All participants were adults and provided written informed consent prior to participation; no minors were enrolled. The study complied with institutional guidelines and the Declaration of Helsinki. All data were anonymized at the point of collection and stored on encrypted institutional servers. De-identified data and materials are publicly available at DOI: https://doi.org/10.6084/m9.figshare.30314464.v3.

#### Experimental design.

This experiment used a 2 × 2 within-participants design. The two factors were Factor A (initial morpheme positional frequency) with two levels (A1: high, A2: low) and Factor B (reading phase) with two levels (B1: learning phase, B2: testing phase). The 2 × 2 within-participants design directly tests both main effects and their interaction with high efficiency and statistical power, and it follows prior Chinese-language studies employing the same paradigm [8,10,37,55,61,72–74]. Each item (trial) comprised six sentences: the first three constituted the learning phase and the subsequent three constituted the testing phase [73,74]. The order of these six sentences was fixed (no within-trial randomization or counterbalancing). To control order effects at the session level, the order of trials (i.e., the six-sentence item blocks) was randomized independently for each participant [73,74].

#### Experimental materials.

**Fig 1:**
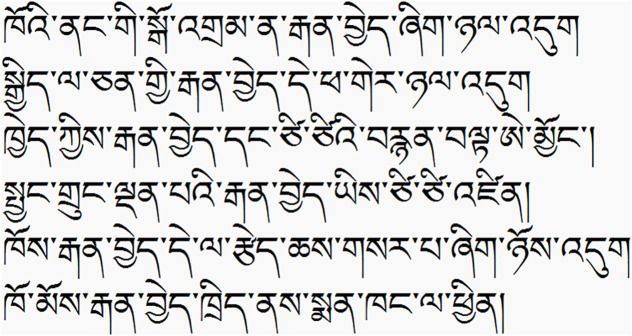
These sentences can be translated to: There is a zopax at his front door. The lazy zopax is sleeping over there. Have you seen cartoons about zopax and mice? The smart zopax caught a mouse. He bought a new toy for the zopax. She took the zopax to the pet hospital. Through these 6 sentences, we can learn that zopax should refer to a type of pet animal, and most likely a type of cat. Figures 1 are original. English glosses are provided for reader clarity.

This study referenced the modern Tibetan corpus created by Robert Barnett et al. [75]. This corpus was extracted from Tibetan newspapers published in recent years and has been processed with word segmentation. We conducted a statistical analysis of all words in this corpus and selected 11 morphemes with high initial morpheme positional frequency and low final morpheme positional frequency (the probability of these morphemes appearing at the beginning of a word exceeds 85%, and the probability of appearing at the end is less than 15%); these were designated as Morpheme 1, following conventions adopted in prior Chinese-language studies on morpheme positional frequency [73,74]. We also selected 10 morphemes with low initial morpheme positional frequency and high final morpheme positional frequency (the probability of these morphemes appearing at the beginning of a word is less than 15%, and the probability of appearing at the end exceeds 85%); these were designated as Morpheme 3. Simultaneously, we selected 15 morphemes with comparable initial and final morpheme positional frequencies (the probability of these morphemes appearing at the beginning and end of a word was between 45% and 55%); these were designated as Morpheme 2. We also controlled for the frequency of these morphemes in Tibetan texts and matched the length of the novel words by character count. Finally, we combined four Morpheme 1s with four Morpheme 2s to create four Tibetan novel words, and combined four Morpheme 3s with four Morpheme 2s to create another four Tibetan novel words. Both sets of novel words used the same Morpheme 2s. The order within each novel word was fixed as M1–M2 (placing M1 word-initial) and M2–M3 (placing M3 word-final), respectively. The same set of Morpheme 2s appeared word-final in the M1–M2 items and word-initial in the M2–M3 items. These novel words were evaluated by 10 native Tibetan speakers (who did not participate in the subsequent experiment) to confirm that they had no actual meaning. They performed a binary real-word judgment, and there was 100% consensus that all items had no attested meaning. Subsequently, each novel word was embedded in six highly constraining sentences. Sentence constraint was established by expert judgment, consistent with conventions in related Chinese-language studies [73,74]. In highly constraining sentences, participants could infer the meaning of the novel word from the sentence context. This process generated eight sets of sentences, with each set containing six sentences. We present an example (one set of sentences) in Fig 1. The procedure for creating our experimental materials was consistent with previous studies [73,74].

**Fig 2:**
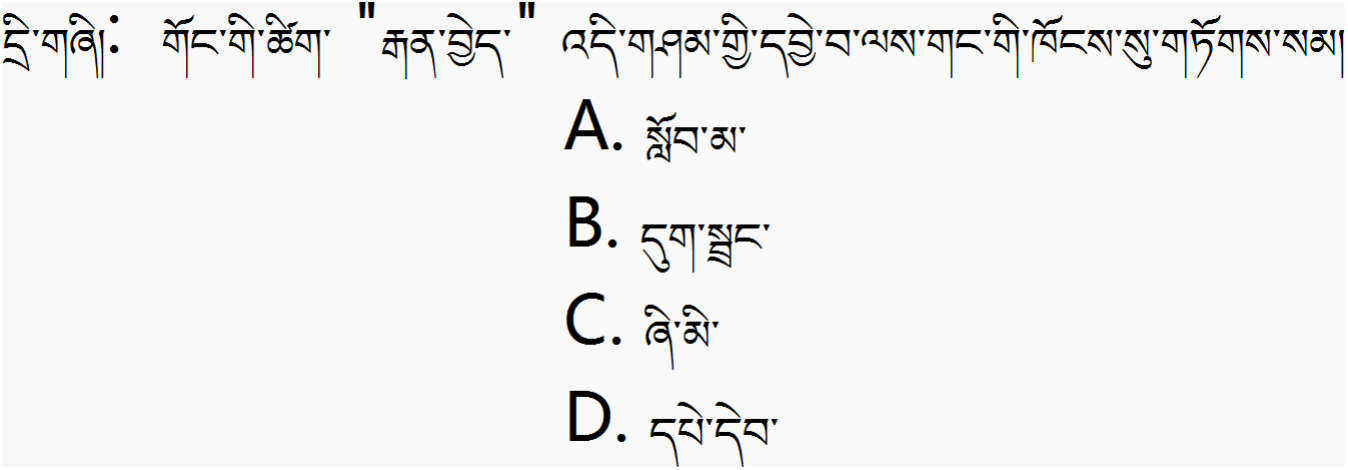
On this image, there is a multiple-choice question. The question part is: The word “ zopax” most likely belongs to which of the following categories? The four options are: A. student, B. insect, C. cat, D. book. Based on reading and inferring from the previous 6 sentences, we can determine that the correct answer is C. Fig 2 are original. English glosses are provided for reader clarity.

Each set of sentences was paired with a corresponding multiple-choice question to test whether the participant truly understood the novel word. An example of the multiple-choice question can be seen in Fig 2.

#### Apparatus.

We used a Tobii X3-120 eye-tracker, which has a sampling frequency of 120 Hz [76]. The eye-tracker was equipped with Tobii Pro Lab, a professional software for designing eye-tracking experiments [76]. We used Tobii Pro Lab to design and conduct the experiment. Stimuli were presented on a 15-inch DELL OLED display with a resolution of 1920 × 1080 pixels. The monitor was mounted on an adjustable stand. A height-adjustable headrest was used to stabilize the participant’s head at a distance of 60 cm from the screen. The laboratory was soundproofed and light-isolated.

#### Procedure.

**Fig 3:**
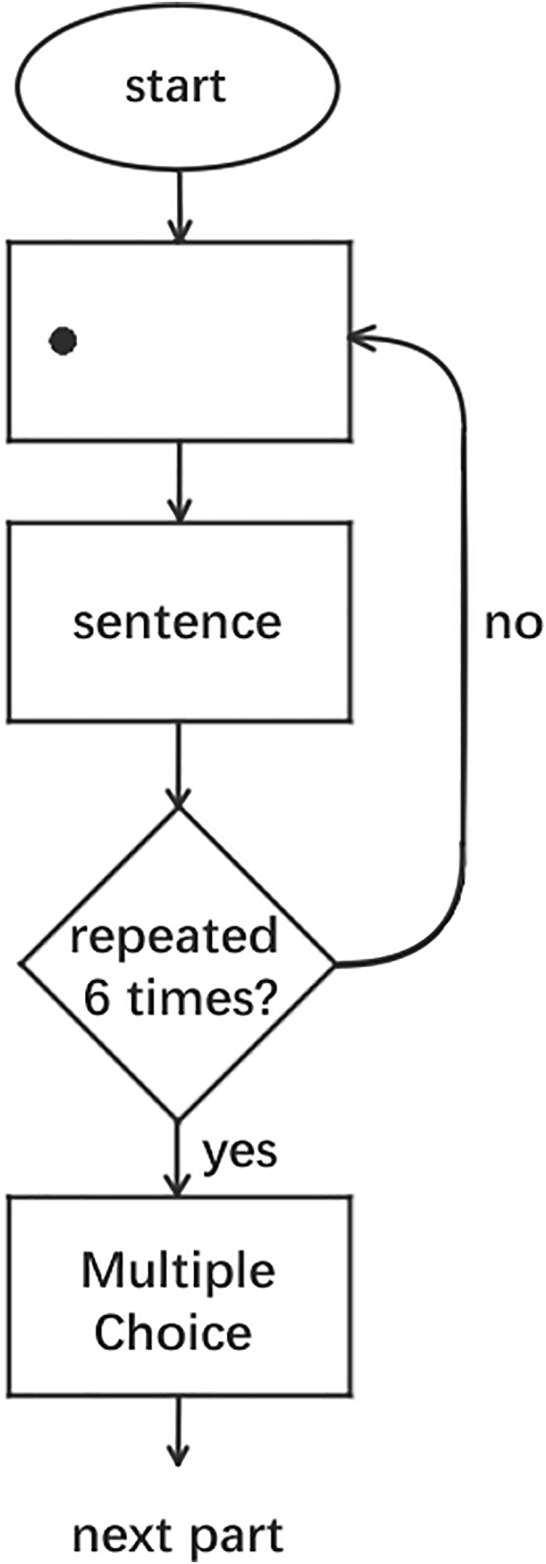
The flowchart of the experimental procedure.

Participants were tested individually. First, a five-point calibration was performed, with an average error of less than 0.3°. After successful calibration, instructions were presented. Participants proceeded to the practice trials after confirming they understood the task. The practice phase comprised 7 screens (6 sentences + 1 multiple-choice question). The formal experiment then began. Per participant, the formal phase included 8 sets × 6 sentences (48 reading screens) plus 8 multiple-choice questions (total 56 screens). One sentence was presented per screen. After reading, participants pressed the spacebar to advance to the next page. Participants read the sentences set by set. Within each set, the order of sentences was fixed. However, the presentation order of the sets was randomized. Following previous studies, we defined the reading of the first three sentences in a set as the learning phase and the reading of the last three sentences as the testing phase [8,61]. After reading a set of sentences, participants were presented with a multiple-choice question. This question required them to determine the semantic category of the learned novel word based on the descriptions in the six sentences. Participants answered the multiple-choice questions on the screen using the Right Ctrl, Left Arrow, Down Arrow, and Right Arrow keys to represent A, B, C, and D, respectively. The Right Ctrl, Left Arrow, Down Arrow, and Right Arrow keys are four consecutive keys on our keyboard; this layout was chosen simply for the convenience of the participants and had no special significance. Only accuracy was analyzed; response times for these multiple-choice questions were not modeled. After answering, participants proceeded to the next set of sentences. The experiment concluded after all sentences were read. A flowchart of the experimental procedure is shown in Fig 3. No additional attention checks were administered beyond the built-in comprehension questions. The entire experiment lasted approximately 8 minutes on average.

#### Measures.

Based on previous research on the acquisition of novel words, we identified three categories of sensitive measures in this field. The first category includes measures reflecting saccade target selection, such as skipping rate and initial fixation position [56,77]. Skipping rate represents the percentage of trials in which a participant did not fixate on the target word during a single sentence presentation [56,77]. Initial fixation position refers to the horizontal position of the first fixation landing within the target word [56,77]. To eliminate the influence of Tibetan word length differences, we converted the horizontal distance of this landing point from the word’s left boundary into a percentage of the word’s width (0% = beginning of the word, 100% = end of the word). These two measures can reflect the participant’s detection and acquisition of morpheme positional frequency in the parafoveal region [56].

The second category includes measures reflecting the early stage of lexical recognition, such as first fixation duration and gaze duration [57,77]. First fixation duration is the duration of the first fixation on the target word [56,77]. It is an indicator of the very initial stage of lexical recognition [57,77]. Gaze duration is the sum of fixation durations on the target word from the first fixation until the gaze first moves away from it [56,77]. It reflects the time required for the initial processing of the target word [57,77].

The third category includes measures reflecting the later stage of lexical recognition, such as number of fixations, total fixation time, and regression pass reading time [73,77]. Number of fixations is the count of fixations on the target word, reflecting the overall processing difficulty of the target word [77,78]. Total fixation time is the sum of all fixation durations on the target word. Compared to gaze duration, total fixation time includes fixations made when the participant returns to the target word after briefly looking away. Therefore, it accounts for instances where the participant experiences confusion after reading past the target word and has to return to it [77,79]. Regression pass reading time is the sum of all fixation durations from the first fixation on the target word until the gaze last moves to the right of it. This measure includes not only fixations on the target word but also fixations to the left of it after the first fixation [77,79].

In summary, we used a total of seven measures in our experiment, allowing for a more comprehensive analysis of participants’ eye movement behavior. For all time-based measures, we coded trials without a fixation on the target area (TOI) as missing (rather than 0) and excluded them from the time-based analyses, consistent with the standard definitions of these measures and common practice in Chinese reading research [73,74,77]. For regression pass reading time, we likewise excluded trials in which the target was skipped on the first pass and only fixated after a regressive saccade, consistent with the standard definitions of these measures and common practice in Chinese reading research [73,74,77]. We acknowledge that these exclusions may introduce bias when skipping differs by condition.

#### Results.

The average answer accuracy was 98.5%, indicating that the participants carefully read and understood the novel words and sentences. Following the existing studies [8,61], data were excluded based on the following three criteria: (1) premature or incorrect key presses that interrupted sentence presentation, (2) invalid data due to loss of tracking, (3) fixation durations of less than 0.08s. Consequently, 0.1% of total eye-movement data points (fixations) in the analysis were excluded due to being invalid. Before analysis, we used the differential evolution algorithm from Python’s SciPy library to calibrate the data of participants by optimizing per-participant calibration to reduce residual drift [80]. To assess robustness, we re-fit all models with versus without the 0.1% flagged fixations; the direction and statistical significance of the fixed effects were unchanged. The raw and processed data needed to reproduce this analysis are available at DOI: https://doi.org/10.6084/m9.figshare.30314464.v3.

Data analysis was conducted in the R environment (version 4.4.3). For continuous dependent variables (first fixation duration, gaze duration, number of fixations, total fixation time, regression pass reading time, and initial fixation position), we fitted Linear Mixed-Effects Models (LMMs) using the lme4 [71] and lmerTest packages. The models included fixed effects for morpheme positional frequency (Factor A: two levels), reading phase (Factor B: two levels), and their interaction. Random intercepts for participants were included as random effects: DV ~ FactorA * FactorB + (1|Recording). P-values and degrees of freedom were estimated using the Satterthwaite approximation implemented in lmerTest. For binary dependent variables (skipping rate), we fitted Generalized Linear Mixed-Effects Models (GLMMs) with a binomial distribution and logit link function, using the same fixed and random effects structure: Binary_DV ~ FactorA * FactorB + (1|Recording). Significance tests for GLMMs were conducted using Type III Wald chi-square tests via the Anova() function from the car package. Models were fitted using the bobyqa optimizer with a maximum of 200,000 function evaluations. Although we report raw means and standard deviations in tables for descriptive purposes, statistical inferences were based on the mixed-effects models described above. We did not apply corrections for multiple comparisons across the seven dependent variables, as each measure was analyzed separately and reflects distinct stages of lexical processing with theoretically motivated predictions.

**Fig 4:**
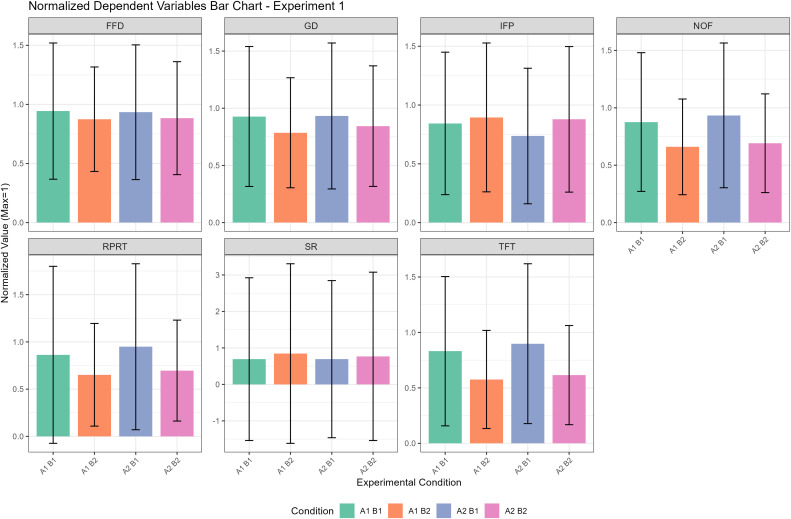
Normalized mean values of dependent variables across experimental conditions in Experiment 1. Each panel represents a different dependent variable (abbreviated as shown in panel headers). Bars show mean values normalized to the maximum value within each variable across both experiments (maximum = 1.0), enabling direct comparison between Experiments 1 and 2. Error bars represent ±1 standard deviation after normalization. Four experimental conditions are shown: A1B1, A1B2, A2B1, and A2B2, representing the factorial combination of two factors (A and B) at two levels each.

To make the experimental results clearer, we have compiled the findings of this experiment into a descriptive statistics table as shown in Table 1, a significance test table as shown in Table 2, and a column chart as illustrated in Fig 4.

**Table 1:** Means and standard deviations of eye movement indicators in experiment 1 (standard deviations in parentheses).

Indicator	A1B1	A1B2	A2B1	A2B2
**Skipping Rate**	0.09(0.29)	0.11(0.32)	0.09(0.28)	0.10(0.30)
**Initial Fixation Position**	27.16(19.51)	28.80(20.38)	23.71(18.56)	28.27(19.93)
**First Fixation Duration**	244.77(149.56)	227.00(114.68)	242.32(147.92)	229.29(124.05)
**Gaze Duration**	344.20(227.14)	291.43(178.51)	345.92(236.74)	312.95(195.59)
**Number of Fixations**	3.32(2.29)	2.50(1.58)	3.54(2.39)	2.62(1.63)
**Total Fixation Time**	810.38(656.47)	561.76(431.45)	876.45(702.86)	599.96(435.92)
**Regression Pass Reading Time**	792.97(860.61)	598.83(499.00)	871.61(806.93)	639.78(490.73)

**Table 2:** Statistical analysis of eye movement indicators in experiment 1. * p < 0.05; ** p < 0.01; *** p < 0.001.

Indicator	Estimate	Std. Error		df	t or Chisq	Pr
skipping rate						
(Intercept)	100.588				100.588***	0.000
FactorA	1.382				1.382	0.240
FactorB	5.007				5.007*	0.025
FactorA:FactorB	1.483				1.483	0.223
initial fixation position						
(Intercept)	27.747	1.143	114.964		24.270***	0.000
factor A	−3.405	1.073	2346.839		−3.172**	0.002
factor B	1.749	1.077	2341.756		1.623	0.105
factor A:factor B	2.566	1.524	2342.487		1.685	0.092
first fixation duration						
(Intercept)	242.837	7.194	145.732		33.754***	0.000
factor A	−1.369	7.513	2348.53		−0.182	0.855
factor B	−18.385	7.542	2342.575		−2.438*	0.015
factor A:factor B	5.215	10.666	2343.545		0.489	0.625
gaze duration						
(Intercept)	338.093	12.199	119.698		27.714***	0.000
factor A	3.241	11.562	2348.098		0.28	0.779
factor B	−53.439	11.605	2343.052		−4.605***	0.000
factor A:factor B	21.244	16.414	2343.785		1.294	0.196
number of fixations						
(Intercept)	3.229	0.137	87.677		23.558***	0.000
factor A	0.23	0.106	2345.258		2.170*	0.030
factor B	−0.824	0.106	2341.62		−7.754***	0.000
factor A:factor B	−0.074	0.15	2342.07		−0.493	0.622
total fixation time						
(Intercept)	782.64	40.68	83.69		19.239***	0.000
factor A	70.6	29.59	2345.59		2.386*	0.017
factor B	−251.23	29.69	2342.38		−8.462***	0.000
factor A:factor B	−21.78	41.99	2342.76		−0.519	0.604
regression pass reading time						
(Intercept)	766.76	42.98	107.84		17.840***	0.000
factor A	87.25	38.39	2169.34		2.273*	0.023
factor B	−192.81	39.04	2165.51		−4.939***	0.000
factor A:factor B	−35.01	55.07	2165.55		−0.636	0.525

Our analysis revealed significant main effects for both initial morpheme positional frequency (factor A) and reading phase (factor B), but no significant interaction between them across all eye-tracking measures.

The main effect of initial morpheme positional frequency (factor A) was significant, primarily influencing later-stage processing and eye movement targeting. Specifically, novel words with a low initial morpheme positional frequency were more difficult to process. This difficulty manifested as a more leftward initial fixation position (*p* = .002), a greater number of fixations (*p* = .030), longer total fixation time (*p* = .017), and longer regression pass reading time (*p* = .023) compared to words with a high-frequency initial morpheme. Notably, this factor did not significantly affect early processing measures like first fixation duration or gaze duration.

The main effect of reading phase (factor B) was also significant, indicating a clear learning effect. As participants moved from the learning phase to the testing phase, their processing of the novel words became significantly more efficient. This was reflected in a higher skipping rate (*p* = .025), shorter first fixation duration (*p* = .015), shorter gaze duration (*p* < .001), fewer number of fixations (*p* < .001), shorter total fixation time (*p* < .001), and shorter regression pass reading time (*p* < .001). However, this factor did not have a significant effect on the measure of initial fixation position.

Finally, except for the initial fixation position showing a marginally significant interaction effect (p = .092), all other indicators showed no significant interaction effects between initial morpheme positional frequency and reading phase (all p > 0.1), suggesting largely independent contributions of positional frequency and learning phase to eye-movement behavior.

## Experiment 2

### Methods

#### Participants.

As in Experiment 1, 56 valid participants were drawn from the same pool as in Experiment 1: 56 of the 60 individuals recruited for Experiment 1 took part in Experiment 2. Four participants with poor gaze-tracking quality in Experiment 1 (valid sample rate < 85%, approximately 30–40%) were not invited back for Experiment 2; no additional exclusions were made. The order of participation (Experiment 1 first vs. Experiment 2 first) was randomized by lot, with a one-week interval between sessions. Given this counterbalancing and the washout interval, potential repeated-exposure and order effects are expected to be minimized and to balance out across participants. Recruitment procedures, inclusion criteria, and demographics matched those of Experiment 1.. All participants signed an informed consent form before the experiment and received 50 RMB as compensation after the experiment.

#### Ethics statement.

This prospective study was approved by the university-level Science and Technology Ethics Committee, Shanxi Normal University (Approval No. 2025−0701, Date: July 20, 2025). Participant recruitment started on 20 July 2025 and ended on 30 July 2025, and all study procedures were conducted at Shanxi Normal University. All participants were adults and provided written informed consent prior to participation; no minors were enrolled. The study complied with institutional guidelines and the Declaration of Helsinki. All data were anonymized at the point of collection and stored on encrypted institutional servers. De-identified data and materials are publicly available at DOI: https://doi.org/10.6084/m9.figshare.30314464.v3. (The same approval covered both experiments.)

#### Experimental Design.

This experiment used a 2 × 2 within-participants design. The two factors were Factor A (final morpheme positional frequency) with two levels (A1: high, A2: low) and Factor B (reading phase) with two levels (B1: learning phase, B2: testing phase). The 2 × 2 within-participants design directly tests both main effects and their interaction with high efficiency and statistical power, and it follows prior Chinese-language studies employing the same paradigm [8,10,37,55,61,72–74]. Each item (trial) comprised six sentences: the first three constituted the learning phase and the subsequent three constituted the testing phase [73,74]. The order of these six sentences was fixed (no within-trial randomization or counterbalancing). To control order effects at the session level, the order of trials (i.e., the six-sentence item blocks) was randomized independently for each participant [73,74].

#### Experimental Materials.

As in Experiment 1, this study referenced the modern Tibetan corpus created by Robert Barnett et al. [75]. This corpus was extracted from Tibetan newspapers published in recent years and has been processed with word segmentation. We conducted a statistical analysis of all words in this corpus and selected 11 morphemes with high initial morpheme positional frequency and low final morpheme positional frequency (the probability of these morphemes appearing at the beginning of a word exceeds 85%, and the probability of appearing at the end is less than 15%); these were designated as Morpheme 1, following conventions adopted in prior Chinese-language studies on morpheme positional frequency [73,74].. We also selected 10 morphemes with low initial morpheme positional frequency and high final morpheme positional frequency (the probability of these morphemes appearing at the beginning of a word is less than 15%, and the probability of appearing at the end exceeds 85%); these were designated as Morpheme 3. Simultaneously, we selected 15 morphemes with comparable initial and final morpheme positional frequencies (the probability of these morphemes appearing at the beginning and end of a word was between 45% and 55%); these were designated as Morpheme 2. We also controlled for the frequency of these morphemes in Tibetan texts and matched the length of the novel words by character count. Finally, we combined four Morpheme 2s with four Morpheme 3s to create four Tibetan novel words, and combined four Morpheme 2s with four Morpheme 1s to create another four Tibetan novel words. Both sets of novel words used the same Morpheme 2s. The order within each novel word was fixed as M1–M2 (placing M1 word-initial) and M2–M3 (placing M3 word-final), respectively. The same set of Morpheme 2s appeared word-final in the M1–M2 items and word-initial in the M2–M3 items. These novel words were evaluated by 10 native Tibetan speakers (who did not participate in the subsequent experiment) to confirm that they had no actual meaning. They performed a binary real-word judgment, and there was 100% consensus that all items had no attested meaning. Subsequently, each novel word was embedded in six highly constraining sentences. Sentence constraint was established by expert judgment, consistent with conventions in related Chinese-language studies [73,74]. In highly constraining sentences, participants could infer the meaning of the novel word from the sentence context. This process generated eight sets of sentences, with each set containing six sentences. The morphemes, novel words, and sentences used in Experiment 2 were all different from those in Experiment 1. The procedure for creating our experimental materials was consistent with previous studies [73,74].

#### Apparatus.

Same as in Experiment 1.

#### Procedure.

Same as in Experiment 1.

#### Measures.

Same as in Experiment 1.

### Results

The average answer accuracy was 98.0%, indicating that the participants carefully read and understood the novel words and sentences. Following the existing studies [8,61], data were excluded based on the following three criteria: (1) premature or incorrect key presses that interrupted sentence presentation, (2) invalid data due to loss of tracking, (3) fixation durations of less than 0.08s. Consequently, less than 0.1% of total eye-movement data points (fixations) in the analysis were excluded due to being invalid. Before analysis, we used the differential evolution algorithm from Python’s SciPy library to calibrate the data of participants by optimizing per-participant calibration to reduce residual drift [80]. To assess robustness, we re-fit all models with versus without the 0.1% flagged fixations; the direction and statistical significance of the fixed effects were unchanged. The raw and processed data needed to reproduce this analysis are available at DOI: https://doi.org/10.6084/m9.figshare.30314464.v3.

Data analysis was conducted in the R environment (version 4.4.3). For continuous dependent variables (first fixation duration, gaze duration, number of fixations, total fixation time, regression pass reading time, and initial fixation position), we fitted Linear Mixed-Effects Models (LMMs) using the lme4 [71] and lmerTest packages. The models included fixed effects for morpheme positional frequency (Factor A: two levels), reading phase (Factor B: two levels), and their interaction. Random intercepts for participants were included as random effects: DV ~ FactorA * FactorB + (1|Recording). P-values and degrees of freedom were estimated using the Satterthwaite approximation implemented in lmerTest. For binary dependent variables (skipping rate), we fitted Generalized Linear Mixed-Effects Models (GLMMs) with a binomial distribution and logit link function, using the same fixed and random effects structure: Binary_DV ~ FactorA * FactorB + (1|Recording). Significance tests for GLMMs were conducted using Type III Wald chi-square tests via the Anova() function from the car package. Models were fitted using the bobyqa optimizer with a maximum of 200,000 function evaluations. Although we report raw means and standard deviations in tables for descriptive purposes, statistical inferences were based on the mixed-effects models described above. We did not apply corrections for multiple comparisons across the seven dependent variables, as each measure was analyzed separately and reflects distinct stages of lexical processing with theoretically motivated predictions.

**Fig 5:**
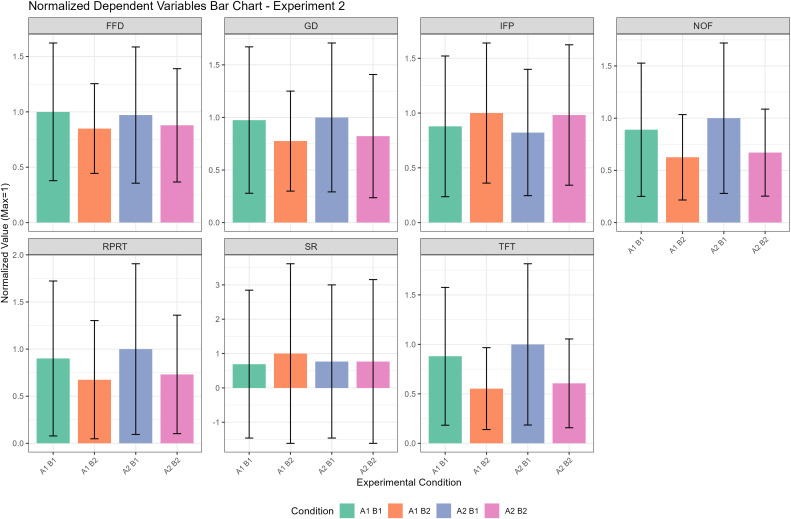
Normalized mean values of dependent variables across experimental conditions in Experiment 2. Data presentation follows the same format as Fig 4. Normalization was performed using the same reference maximum values as in Fig 1 (i.e., the maximum mean value for each variable across both experiments combined), allowing for direct quantitative comparison of effect magnitudes between the two experimental replications.

To make the experimental results clearer, we have compiled the findings of this experiment into a descriptive statistics table as shown in Table 3, a significance test table as shown in Table 4, and a column chart as illustrated in Fig 5.

**Table 3:** Means and standard deviations of eye movement indicators in experiment 2 (standard deviations in parentheses).

Indicator	A1B1	A1B2	A2B1	A2B2
**Skipping Rate**	0.09(0.28)	0.13(0.34)	0.10(0.29)	0.10(0.31)
**Initial Fixation Position**	28.28(20.68)	32.19(20.59)	26.48(18.57)	31.62(20.63)
**First Fixation Duration**	259.50(161.32)	220.44(105.12)	251.91(159.61)	227.84(132.77)
**Gaze Duration**	361.75(258.28)	287.46(176.45)	370.95(262.66)	305.12(217.45)
**Number of Fixations**	3.37(2.42)	2.37(1.55)	3.79(2.73)	2.54(1.58)
**Total Fixation Time**	857.86(679.25)	539.49(403.54)	975.36(794.77)	591.21(437.60)
**Regression Pass Reading Time**	827.25(755.12)	620.54(576.13)	918.71(831.82)	671.84(577.59)

**Table 4:** Statistical analysis of eye movement indicators in experiment 2. * p < 0.05; ** p < 0.01; *** p < 0.001.

Indicator	Estimate	Std. Error		df	t or Chisq	Pr
skipping rate						
(Intercept)	111.277				111.277***	0.000
FactorA	0.094				0.094	0.759
FactorB	13.277				13.277***	0.000
FactorA:FactorB	2.835				2.835	0.092
initial fixation position						
(Intercept)	28.937	1.154	115.72		25.078***	0.000
factor A	−1.752	1.109	2320.456		−1.58	0.114
factor B	3.799	1.12	2324.44		3.393***	0.001
factor A:factor B	1.295	1.579	2320.521		0.82	0.412
first fixation duration						
(Intercept)	258.478	8.422	109.713		30.693***	0.000
factor A	−7.707	7.736	2321.977		−0.996	0.319
factor B	−39.488	7.814	2325.479		−5.054***	0.000
factor A:factor B	16.163	11.019	2322.021		1.467	0.143
gaze duration						
(Intercept)	356.852	14.485	102.679		24.635***	0.000
factor A	9.204	12.473	2323.747		0.738	0.461
factor B	−74.77	12.6	2326.685		−5.934***	0.000
factor A:factor B	9.5	17.767	2323.776		0.535	0.593
number of fixations						
(Intercept)	3.28	0.142	90.418		23.077***	0.000
factor A	0.412	0.114	2321.856		3.626***	0.000
factor B	−0.982	0.115	2324.443		−8.551***	0.000
factor A:factor B	−0.259	0.162	2321.878		−1.6	0.110
total fixation time						
(Intercept)	832.22	43.13	83.12		19.298***	0.000
factor A	114.5	31.27	2322.68		3.662***	0.000
factor B	−314.74	31.59	2324.75		−9.965***	0.000
factor A:factor B	−66.03	44.53	2322.7		−1.483	0.138
regression pass reading time						
(Intercept)	805.7	45.57	98.52		17.682***	0.000
factor A	83.9	38.47	2143.73		2.181*	0.029
factor B	−200.17	39.38	2147.19		−5.083***	0.000
factor A:factor B	−40.08	55.34	2144.2		−0.724	0.469

Our analysis of the final morpheme positional frequency experiment revealed significant main effects for both final morpheme positional frequency (factor A) and reading phase (factor B) on several measures. However, no significant interaction was found between the two factors on any eye-tracking metric.

The main effect of final morpheme positional frequency (factor A) was significant on later-stage processing measures. Novel words with a high final morpheme positional frequency were processed more easily, receiving a fewer number of fixations (p < .001), a shorter total fixation time (p < .001), and a shorter regression pass reading time (p = .029). Unlike the initial morpheme positional frequency experiment (Experiment 1), this factor did not significantly affect eye movement targeting (initial fixation position and skipping rate) or any early processing measures, including first fixation duration and gaze duration.

The main effect of reading phase (factor B) demonstrated a clear learning effect, consistent with the previous experiment. As participants moved from the learning to the testing phase, processing became more efficient. This was reflected in a significantly higher skipping rate (p < .001), a more rightward initial fixation position (p < .001), shorter first fixation duration (p < .001), shorter gaze duration (p < .001), fewer number of fixations (p < .001), shorter total fixation time (p < .001), and shorter regression pass reading time (p < .001).

Finally, except for the skipping rate showing a marginally significant interaction effect (p = .092), all other indicators showed no significant interaction effects between final morpheme positional frequency and reading phase (all p > 0.1), indicating that the effects of final morpheme positional frequency and reading phase were independent of each other.

## Discussion

### Differences between the reading phase and morpheme positional frequency

From the significance tables (Table 2 and Table 4) and the column charts (Figures 4 and 5), the impact of the reading phase appears much larger than that of morpheme positional frequency across measures. Across the two experiments, only initial fixation position in Experiment 1 showed a significant effect of initial morpheme positional frequency (Factor A) but a non-significant effect of the reading phase (Factor B). For all other measures without exception, both the significance level (p value) and the estimate values (effect sizes) for the reading phase was stronger than that for morpheme positional frequency. This pattern is consistent with prior work in Chinese [8,61,73]. The discrepancy can be explained by the processing level and scope at which the two variables operate.

First, the reading phase is a macro-level, task-level variable that produces a global, top-down facilitation effect. On a first pass, readers must allocate substantial cognitive resources to lexical access, syntactic parsing, and semantic integration [81,82]. Re-reading, by contrast, triggers strong familiarity effects and repetition priming, reducing the overall cognitive load of the text and shifting processing from relatively controlled to highly automatic [83–85]. As a result, eye-movement behavior undergoes global and pronounced changes (e.g., shorter fixation durations and fewer regressions), an effect that is overwhelming.

Second, morpheme positional frequency is a micro-level, stimulus-level variable that operates during bottom-up lexical processing. This statistical regularity is part of readers’ implicit knowledge of language and is used unconsciously and automatically. It likely exerts fine-grained, local modulation at specific stages of lexical recognition, such as orthographic processing or word segmentation [86–88]. Its role is to fine-tune an existing processing stream.

Hence, the stark difference in effect sizes (the estimate values) reflects a fundamental contrast between global changes in task state and local adjustments to stimulus features. The former alters how readers interact with the entire set of experimental materials, whereas the latter plays a subtle, millisecond-scale modulatory role within lexical processing. Its influence is naturally much smaller than that of the reading phase.

### Commonalities of initial and final morpheme positional frequency

For early measures of lexical recognition, neither initial morpheme positional frequency nor final morpheme positional frequency showed significant effects. This contrasts slightly with some studies in Chinese, where marginal effects have occasionally appeared on gaze duration [8,61,73]. Although we follow the common practice of treating gaze duration as an early measure, its classification as an early versus middle measure remains debated [61,89]. The complete absence of effects in our study strengthens the conclusion that morpheme positional frequency does not produce differences at the early stage of lexical recognition in Tibetan. By contrast, for late measures of lexical recognition, both initial morpheme positional frequency and final morpheme positional frequency showed significant effects. We infer that, in Tibetan, during the acquisition of novel words, both initial and final morpheme positional frequency influence late-stage lexical recognition. This conclusion is consistent with findings from the acquisition of novel words in Chinese [8,61,73]. The absence of early effects in Tibetan carries important theoretical implications for cross-linguistic models of word recognition. First, it suggests that morpheme positional frequency operates as a segmentation cue during post-lexical integration rather than during initial lexical access. Second, the convergence between Tibetan (a phonographic, delimiter-marked script) and Chinese (a logographic, unspaced script) on this temporal profile indicates that certain aspects of word learning may be universal despite surface differences in orthographic structure [8, 61, 73]. This cross-linguistic consistency supports models that posit distinct processing stages for lexical access versus lexical integration, with statistical segmentation cues like positional frequency primarily influencing the latter stage.

With the exception of the initial fixation position measure in Experiment 1 and the skipping rate measure in Experiment 2, interactions between morpheme positional frequency and the reading phase were absent across all other measures—another commonality of initial and final morpheme positional frequency. The lack of interaction suggests that, at both early and late stages of lexical recognition, morpheme positional frequency and the reading phase operate independently. This diverges from research in Chinese. In studies on the acquisition of novel words in Chinese, robust interactions are commonly reported for Regression Pass Reading Time and number of fixations, and sometimes also for measures such as gaze duration and total fixation time [8,61,73]. The widespread inconsistency across measures suggests that Chinese and Tibetan likely differ in some property that eliminates the interaction between morpheme positional frequency and the reading phase in Tibetan. Whether that property is the morpheme delimiter “་” in Tibetan, Tibetan’s phonographic nature, or something else remains to be determined.

### Differences between initial and final morpheme positional frequency

Although initial and final morpheme positional frequency exerted broadly similar influences on early and late lexical recognition, the magnitudes of their effects may differ. Among the late measures that did show effects, final morpheme positional frequency appeared to more strongly affect number of fixations and total fixation time, whereas the effects of initial and final morpheme positional frequency on Regression Pass Reading Time appeared comparable. This pattern is understandable: while all three measures are late measures, Regression Pass Reading Time is more sensitive to regressions triggered by the target word itself, whereas number of fixations and total fixation time are more sensitive to regressions from post-target regions back to the target word [77,79]. Differences in what each measure is most sensitive to likely produced the observed dissociations. This differs from prior research in Chinese. In Chinese natural reading, only final morpheme positional frequency significantly affects word segmentation and lexical recognition, whereas initial morpheme positional frequency does not [55]. That pattern is more similar to our findings for number of fixations and total fixation time. In Chinese research on the acquisition of novel words, the effects of initial and final morpheme positional frequency on late-stage recognition measures are similar, with no clear dominance of one over the other [8,61,73]. That pattern is more similar to our findings for Regression Pass Reading Time.

One tentative hypothesis for this discrepancy is that Tibetan morphemes, compared with Chinese characters, may provide stronger cues to word boundaries due to differences in script structure and morphological transparency. For example, in English text without spaces, encountering the string “ing” typically suggests a suffix of the preceding word; if “ing” happened to be the beginning of an unknown word, readers would likely regress to check that it is not the preceding word’s suffix before treating it as the onset of a novel word. Similarly, a morpheme like “pre” would likely prompt a forward look-ahead to confirm its status as a word-initial element. Such bidirectional checks could potentially amplify the influence of both initial and final morpheme positional frequency. While this analogy is illustrative, Tibetan indeed has word-formation rules analogous to English, while Chinese does not. This could potentially explain why initial morpheme positional frequency is not significant in Chinese natural reading but is significant in Tibetan reading. However, this remains a speculative interpretation that requires empirical validation. As for why final morpheme positional frequency produces stronger effects than initial morpheme positional frequency on number of fixations and total fixation time in our Tibetan data, one possibility is that, by the time readers reach the target word, they have already confirmed the right edge of the preceding word from earlier parts of the sentence, reducing the impact of the target word’s initial morpheme positional frequency. This pattern has also been documented in Chinese, and the same explanation is widely accepted in that literature [55,90].

The differences between initial and final morpheme positional frequency are most apparent in saccade target selection—namely, in skipping rate and initial fixation position. We first consider initial fixation position, the clearest case. In Tibetan during the acquisition of novel words, initial morpheme positional frequency had a significant effect on initial fixation position, suggesting that higher initial morpheme positional frequency may facilitate saccade target selection (or that lower initial morpheme positional frequency may hinder it). In Experiment 1, initial fixation position was the only measure for which the effect of morpheme positional frequency exceeded the effect of the reading phase, and the only measure whose morpheme positional frequency effect was highly significant (p = .002), hinting at a tight linkage between this measure and initial morpheme positional frequency. In Chinese, however, no significant effect on initial fixation position has been reported [8,10,37,55,61,72–74], though methodological factors (see Limitations section) may partially account for this null finding.

Second, for skipping rate, we observed a marginal interaction (p < 0.1) between final morpheme positional frequency and the reading phase, whereas initial morpheme positional frequency showed no such interaction. Specifically, although the level of final morpheme positional frequency did not affect the skipping rate during the first few readings, when final morpheme positional frequency was high, participants appeared more likely to show higher skipping rates in subsequent readings. This is similar to earlier findings in Chinese, where Liang et al. reported a significant effect of final morpheme positional frequency at the 0.05 level during natural reading, but no such effect for initial morpheme positional frequency [55]. Liang et al. did not examine the interaction between final morpheme positional frequency and the reading phase [73]. However, given that most other measures showed no such interaction and this effect only reached marginal significance (p < .1), we are inclined to treat this finding as statistically unreliable, though future research may help clarify whether a genuine interaction exists.

Finally, the interaction between initial morpheme positional frequency and the reading phase on initial fixation position was marginal. Specifically, when initial morpheme positional frequency was high, participants localized the target word well during the learning phase (the first three sentences), whereas when initial morpheme positional frequency was low, good localization emerged only during the testing phase (the last three sentences). This seems to indicate that high initial morpheme positional frequency facilitates target localization. Prior Chinese studies, however, did not examine the interaction between initial morpheme positional frequency and the reading phase on this measure [73]. However, given that this interaction only reached marginal significance (p < .1) and most other measures showed no interaction effects, we tend to consider this finding statistically unreliable and caution against drawing strong conclusions without further replication.

### Limitations and prospects

1. Further verification of the interaction between initial and final morpheme positional frequency and the reading phase in saccade target selection.

Based on our analyses, we found no interaction between morpheme positional frequency (initial and final) and the reading phase at either early or late stage of lexical recognition during the acquisition of novel words in Tibetan. However, for the two measures of saccade target selection, whether morpheme positional frequency (initial and final) interact with the reading phase remains unclear. Relying only on two marginally significant measures and lacking corroborating studies, we cannot determine whether such an interaction truly exists. This remains an open question in Tibetan reading research on morpheme positional frequency that urgently needs to be addressed. Therefore, using different technical methods and reading tasks to further verify whether initial and final morpheme positional frequency interact with the reading phase in saccade target selection is necessary.

2. Examine the impact of morpheme positional frequency on Tibetan word segmentation and Tibetan reading in other tasks.

This study explored how morpheme positional frequency influences Tibetan reading in the acquisition of novel words. However, the novel word learning paradigm is only a standard paradigm for probing morpheme positional frequency. In daily reading, we seldom engage in the acquisition of novel words. Therefore, it is necessary to examine how morpheme positional frequency affects Tibetan word segmentation and Tibetan reading in other reading tasks. In research on Chinese, some scholars investigated the effect of morpheme positional frequency in natural reading, and others examined its effect in overlapping ambiguous strings [55,62]. Still others investigated its effect during oral reading [63], and some specifically examined the effect of morpheme positional frequency in the preview stage [91]. These studies are important, as they offer a comprehensive account of the role of morpheme positional frequency within a language. In addition, most existing research has focused on two-character words. Extending this work to words with more than two characters (morphemes) is a feasible future direction for research on morpheme positional frequency.

3. Interactions between morpheme positional frequency and spaces as low-level word segmentation cues.

Spaces, as a low-level word segmentation cue, have been widely shown to play an important role in word segmentation and lexical recognition. As a segmentation cue as well, is the mechanism and time course of morpheme positional frequency consistent with that of spaces? This can be tested by examining the interaction between morpheme positional frequency and spaces on indices of word segmentation and reading. In research on Chinese, some scholars found that spaces and morpheme positional frequency influence the processes of word segmentation and lexical recognition independently [61]. One proposed explanation is that spaces operate at the early stage of segmentation and recognition, whereas morpheme positional frequency operates at a later stage [61]; therefore, their effects are independent. However, such studies are scarce, and we need to explore this issue in more languages to substantiate this conclusion.

4. Investigate other higher-level word segmentation cues in Tibetan

Morpheme positional frequency is not the only higher-level word segmentation cue. Factors such as lexical familiarity, word frequency, and lexical plausibility may all affect the process of word segmentation. Previous studies demonstrated that, in Chinese, lexical familiarity, word frequency, and lexical plausibility can affect word segmentation [17,34]. However, more fine-grained, in-depth research is lacking. In the review by Gao and colleagues, exploring other higher-level word segmentation cues was explicitly identified as a future direction for research on Chinese [36]. In Tibetan, do higher-level segmentation cues similar to those in Chinese exist? What are the underlying mechanisms of these cues? Is the time course of different higher-level cues consistent? Do different higher-level cues interact with one another? These questions require more detailed and further research.

5. Methodological considerations in eye-tracking calibration

A notable methodological difference between our study and prior Chinese research concerns eye-tracker calibration procedures. Due to the high spatial precision required for analyzing Tibetan script—where even a 0.3° deviation can misplace a fixation by more than half a character’s width—we implemented a post-hoc, subject-specific calibration based on a Differential Evolution algorithm. This procedure corrects for non-linear calibration drift by optimizing fixation placement within word boundaries using a piecewise linear model. While this approach substantially increased the probability that participants’ fixations landed on words (rather than on inter-word spaces or regions outside the sentence) in our study, it represents a departure from standard calibration protocols.

This divergence has implications for cross-linguistic comparisons. Many Chinese studies on morpheme positional frequency did not report whether validation points differed from calibration points, nor whether post-hoc corrections were applied [8,10,37,55,61,72–74]. If similar calibration drift occurred in those studies—particularly for measures highly sensitive to interest-area boundaries, such as initial fixation position—it could partially explain the null effects reported for initial morpheme positional frequency in Chinese [55,73]. Conversely, our customized calibration procedure, while necessary for Tibetan’s spatial demands, limits direct comparability with studies using standard protocols. Future research employing consistent, high-precision calibration methods (e.g., custom validation procedures or nonlinear corrections) across both writing systems would enable more definitive conclusions about language-specific versus methodological sources of variation in morpheme positional frequency effects.

## Conclusions

Within the specific context of novel word acquisition in Tibetan, this study provides evidence for the role of morpheme positional frequency in word segmentation and lexical recognition. Findings suggest that morpheme positional frequency may function as an important word segmentation cue in Tibetan in this learning paradigm. In the acquisition of novel words in Tibetan, initial morpheme positional frequency does not affect the early stage of lexical recognition, but it does affect saccade target selection and also affects the late stage of lexical recognition. Final morpheme positional frequency has no effect on saccade target selection or on the early stage of lexical recognition, but it does affect the late stage of lexical recognition. The reading phase affects saccade target selection as well as both the early and the late stage of lexical recognition. At both the early and the late stage of lexical recognition, the effects of initial and final morpheme positional frequency and the effects of the reading phase are independent. For saccade target selection, the effects of initial and final morpheme positional frequency may interact with the reading phase, but further verification is required.

It is important to note that these findings are based on a novel word learning paradigm and may not fully generalize to natural Tibetan reading contexts. Further research using different reading tasks and materials is needed to comprehensively understand the role of morpheme positional frequency in Tibetan word segmentation across various reading situations.

## References

[1] Li X, Rayner K, Cave KR. On the segmentation of Chinese words during reading. Cogn Psychol. 2009;58(4):525–52. doi: 10.1016/j.cogpsych.2009.02.003 19345938

[2] Zang C, Liang F, Bai X, Yan G, Liversedge SP. Interword spacing and landing position effects during Chinese reading in children and adults. J Exp Psychol Hum Percept Perform. 2013;39(3):720–34. doi: 10.1037/a0030097 23067120

[3] Rayner K, Fischer MH, Pollatsek A. Unspaced text interferes with both word identification and eye movement control. Vision Res. 1998;38(8):1129–44. doi: 10.1016/s0042-6989(97)00274-5 9666972

[4] Wang D, Niu D, Li T, Gao X. The Effect of Visual Word Segmentation Cues in Tibetan Reading. Brain Sci. 2024;14(10):964. doi: 10.3390/brainsci14100964 39451978 PMC11505889

[5] Ju JX, Zhang XL, Meng LT. Word segmentation basis and eye movement performance in text reading. Journal of Liaoning Normal University (Social Science Edition). 2014;37:211–6.

[6] Bai XJ, Yan GL, Wang JX, Zang CL, Zhu ZH, Tian J, et al. Eye-movement study on word segmentation promoting reading performance of Chinese beginners. Psychological Science Progress. 2012. Available from: https://kns.cnki.net/kcms2/article/abstract?v=TajfHsrud99uOqSIG0vu_5sjRedczLT0hzJmqKwIB-njAkOqYWRF3L4GsuwoB4ZtfvwxAFXDsQRtGaqIfEaQN8ymvcAsAI-3jzhYBPS5aGdHAgetHi25bxTnpIMV4N7dobVsiKYfxFDbx4G4IR6XAaYBFx_axKZ86Z28SlQ9Kcl3MzE-oSWk3A==&uniplatform=NZKPT&language=CHS

[7] Blythe HI, Liang F, Zang C, Wang J, Yan G, Bai X, et al. Inserting spaces into Chinese text helps readers to learn new words: An eye movement study. J Memory Language. 2012;67(2):241–54. doi: 10.1016/j.jml.2012.05.004

[8] Liang F, Blythe HI, Zang C, Bai X, Yan G, Liversedge SP. Positional character frequency and word spacing facilitate the acquisition of novel words during Chinese children’s reading. J Cognitive Psychol. 2015;27(5):594–608. doi: 10.1080/20445911.2014.1000918

[9] Bai XJ, Zhang T, Tian LJ, Liang FF, Wang TL. Eye movement study on the influence of word segmentation on Chinese reading for American overseas students. Psychol Res. 2010;3:25–30.

[10] Liang FF. Eye-movement study on the cognitive mechanism of Chinese word segmentation. Tianjin Normal University. 2013. https://kns.cnki.net/kcms2/article/abstract?v=TajfHsrud9-nYZEK70pZ7WEor7Ayx9t-vJ_vU1psDS3jZNOX5WuQOEcoPgXO2rEEG8-lnKWrPjkZTH7bNcQz3HCWRuOorKl4eddq9LCboxCJ4OdikZP0G1Oqf8J9GdVOB-wy7XTd9nwWdrFYpLQlGM70slUCGJFZ2rgHik6-3TLfs1XfPAtxBA==&uniplatform=NZKPT&language=CHS

[11] CAO H, LAN Z, GAO F, YU H, LI P, WANG J. The role of character positional frequency on word recognition during Chinese reading: Lexical decision and eye movements studies. Acta Psychologica Sinica. 2023;55(2):159. doi: 10.3724/sp.j.1041.2023.00159

[12] Zhao W, Zhou S, Wang TZ. Eye-movement study on the influence of word segmentation on early reading of preschool children. Preschool Education Res. 2015;7:14–21.

[13] Shen D, Liversedge SP, Tian J, Zang C, Cui L, Bai X, et al. Eye movements of second language learners when reading spaced and unspaced Chinese text. J Exp Psychol Appl. 2012;18(2):192–202. doi: 10.1037/a0027485 22545927

[14] Guo ZY, Bai XJ, Gu L, Wang YS, Wang LH. Eye-movement study on the influence of word segmentation on Japanese reading of Japanese-Chinese and Chinese-Japanese bilinguals. Stud Psychol Behavior. 2014;12:775–81.

[15] SHEN D-L, BAI X-J, ZANG C-L, YAN G-L, FENG B-C, FAN X-H. Effect of Word Segmentation on Beginners’ Reading: Evidence from Eye Movements. Acta Psychologica Sinica. 2010;42(2):159–72. doi: 10.3724/sp.j.1041.2010.00159

[16] Ma G, Li X, Rayner K. Word segmentation of overlapping ambiguous strings during Chinese reading. J Exp Psychol Hum Percept Perform. 2014;40(3):1046–59. doi: 10.1037/a0035389 24417292 PMC4214861

[17] Xiang HW, Hu XZ, Yang RR, Sui X. The basis of Chinese word segmentation and the explanation of eye movement control models. Psychol Sci. 2018;41:1097–103.

[18] Yen M-H, Radach R, Tzeng OJ-L, Tsai J-L. Usage of statistical cues for word boundary in reading Chinese sentences. Read Writ. 2011;25(5):1007–29. doi: 10.1007/s11145-011-9321-z

[19] Pollatsek A, Rayner K. Eye movement control in reading: The role of word boundaries. Journal of Experimental Psychology: Human Perception and Performance. 1982;8(6):817–33. doi: 10.1037/0096-1523.8.6.817

[20] Sheridan H, Rayner K, Reingold EM. Unsegmented text delays word identification: Evidence from a survival analysis of fixation durations. Visual Cognition. 2013;21(1):38–60. doi: 10.1080/13506285.2013.767296

[21] Perea M, Acha J. Space information is important for reading. Vision Res. 2009;49(15):1994–2000. doi: 10.1016/j.visres.2009.05.009 19463847

[22] Morris RK, Rayner K, Pollatsek A. Eye movement guidance in reading: the role of parafoveal letter and space information. J Exp Psychol Hum Percept Perform. 1990;16(2):268–81. doi: 10.1037//0096-1523.16.2.268 2142198

[23] Song Z, Wang Y, Liu N, Han Y, Yan G. The effect of alternating-color word boundary on oral and silent reading in Grade 1 Chinese children: Evidence from eye movements. Stud Psychol Behavior. 2021;19(2):172–8.

[24] Yan G, Liang X, Song Z, He S, Lan Z, Dong C. Word segmentation by alternating colors facilitates text reading for senior deaf students in primary school: evidence from eye movements. J Psychological Science. 2019;42(3):570–6.

[25] Inhoff AW, Liu W. The perceptual span and oculomotor activity during the reading of Chinese sentences. J Exp Psychol Hum Percept Perform. 1998;24(1):20–34. doi: 10.1037//0096-1523.24.1.20 9483822

[26] Bai X, Yan G, Liversedge SP, Zang C, Rayner K. Reading spaced and unspaced Chinese text: Evidence from eye movements. J Experimental Psychol: Human Perception and Performance. 2008;34(5):1277–87. 10.1037/0096-1523.34.5.1277 PMC2662925 18823210

[27] Meng SY. The effectiveness of color marking as word segmentation clue in Chinese reading. Tianjin Normal University. 2017. https://kns.cnki.net/kcms2/article/abstract?v=TajfHsrud9_OjdiMyQutLKfW7tRr7ioAG5VYtVOBzOVv92HqIJS_UbV0_bkeFzxRVSWsjaOrjYR-UlpzVSuSG-cNFJlSC3ry9Luqntgv1dMxz_OO6MPHz1jwRj4hg2OvSbYgFhnjZQxC0xCdN7xTHUjE7ozhTe9Jh_iI2MKJNjyIY0bXe5md5g==&uniplatform=NZKPT&language=CHS

[28] Wang DH. The role of visual word segmentation clues in Tibetan reading. Tibet University. 2023. doi: 10.27735/d.cnki.gxzdx.2023.000220

[29] Li XS, Liu PP, Ma GJ. Advances in cognitive mechanisms of word segmentation during Chinese reading. Advances in Psychological Science. 2011;19(4):459–67.

[30] McClelland JL, Rumelhart DE. An interactive activation model of context effects in letter perception: I. An account of basic findings. Psychological Review. 1981;88(5):375–407. doi: 10.1037/0033-295x.88.5.375 7058229

[31] Reichle ED, Pollatsek A, Fisher DL, Rayner K. Toward a model of eye movement control in reading. Psychol Rev. 1998;105(1):125–57. doi: 10.1037/0033-295x.105.1.125 9450374

[32] Rayner K, Li X, Pollatsek A. Extending the e-z reader model of eye movement control to chinese readers. Cogn Sci. 2007;31(6):1021–33. doi: 10.1080/03640210701703824 21635327

[33] Yu WB, Liang DD. Word segmentation clues in spoken language processing. Ad Psychological Sci. 2018;26:1765–74.

[34] Zang C, Wang Y, Bai X, Yan G, Drieghe D, Liversedge SP. The use of probabilistic lexicality cues for word segmentation in Chinese reading. Q J Exp Psychol (Hove). 2016;69(3):548–60. doi: 10.1080/17470218.2015.1061030 26145449

[35] Li Y, Jia YJ, Zong C, Yu H. Research and implementation of Tibetan automatic word segmentation based on conditional random field. J Chinese Information Processing. 2013;27(4):52–8.

[36] Gao XL, Niu DY, Yang XY, Zhang YL. The impact of character positional frequency information on word segmentation in Chinese reading. J Liaoning Normal University (Social Science Edition). 2024;47:53–9.

[37] Liang FF, Wang YS, Yan GL, Bai XJ. The application of word segmentation clues in new word learning: morpheme position probability. In: Eighteenth National Psychology Academic Conference Abstracts Collection—Psychology and Social Development. 2015;336–7.

[38] McGowan VA, White SJ, Paterson KB. The effects of interword spacing on the eye movements of young and older readers. Journal of Cognitive Psychology. 2014;27(5):609–21. doi: 10.1080/20445911.2014.988157

[39] Juhasz BJ, White SJ, Liversedge SP, Rayner K. Eye movements and the use of parafoveal word length information in reading. J Exp Psychol Hum Percept Perform. 2008;34(6):1560–79. doi: 10.1037/a0012319 19045993 PMC2668122

[40] Inhoff AW, Radach R. Definition and Computation of Oculomotor Measures in the Study of Cognitive Processes. Eye Guidance in Reading and Scene Perception. Elsevier. 1998. p. 29–53. doi: 10.1016/b978-008043361-5/50003-1

[41] Veldre A, Andrews S. Lexical quality and eye movements: individual differences in the perceptual span of skilled adult readers. Q J Exp Psychol (Hove). 2014;67(4):703–27. doi: 10.1080/17470218.2013.826258 23972214

[42] Rayner K, Pollatsek A. Eye movements in reading: A tutorial review. In: Coltheart M, editor. Attention and Performance XII. Routledge. 2016. p. 327–62.

[43] McConkie GW, Kerr PW, Reddix MD, Zola D. Eye movement control during reading: I. The location of initial eye fixations on words. Vision Res. 1988;28(10):1107–18. doi: 10.1016/0042-6989(88)90137-x 3257013

[44] Price-Mohr RM, Bernard Price C. Increasing inter-word spacing reduces migration errors and improves reading comprehension in students with dyslexia. Dyslexia. 2024;30(4):e1787. doi: 10.1002/dys.1787 39139062

[45] Bouma H. Interaction effects in parafoveal letter recognition. Nature. 1970;226(5241):177–8. doi: 10.1038/226177a0 5437004

[46] Mirault J, Snell J, Grainger J. Reading without spaces: The role of precise letter order. Atten Percept Psychophys. 2019;81(3):846–60. doi: 10.3758/s13414-018-01648-6 30628036 PMC6407912

[47] Sheridan H, Reichle ED, Reingold EM. Why does removing inter-word spaces produce reading deficits? The role of parafoveal processing. Psychon Bull Rev. 2016;23(5):1543–52. doi: 10.3758/s13423-015-0997-y 26801166

[48] Rayner K, Slattery TJ, Drieghe D, Liversedge SP. Eye movements and word skipping during reading: effects of word length and predictability. J Exp Psychol Hum Percept Perform. 2011;37(2):514–28. doi: 10.1037/a0020990 21463086 PMC3543826

[49] White SJ. Eye movement control during reading: Effects of word frequency and orthographic familiarity. J Exp Psychol Hum Percept Perform. 2008;34(1):205–23. doi: 10.1037/0096-1523.34.1.205 18248149

[50] Toshima M, Ishikawa T, Mogi K. Spaces enhance word segmentation and comprehension it tacit reading. In International Symposium on Neural Networks. Berlin, Heidelberg; Springer. 2011; 76–82.

[51] Sainio M, Hyönä J, Bingushi K, Bertram R. The role of interword spacing in reading Japanese: an eye movement study. Vision Res. 2007;47(20):2575–84. doi: 10.1016/j.visres.2007.05.017 17697693

[52] Kajii N, Nazir TA, Osaka N. Eye movement control in reading unspaced text: the case of the Japanese script. Vision Res. 2001;41(19):2503–10. doi: 10.1016/s0042-6989(01)00132-8 11483180

[53] Fujii A, Ishikawa T. Japanese/English Cross-Language Information Retrieval: Exploration of Query Translation and Transliteration. Compu Humanities. 2001;35(4):389–420. doi: 10.1023/a:1011856202986

[54] Wang H, Legge GE. Comparing the minimum spatial-frequency content for recognizing Chinese and alphabet characters. J Vis. 2018;18(1):1. doi: 10.1167/18.1.1 29297056 PMC5749648

[55] Liang F, Gao Q, Li X, Wang Y, Bai X, Liversedge SP. The importance of the positional probability of word final (but not word initial) characters for word segmentation and identification in children and adults’ natural Chinese reading. J Exp Psychol Learn Mem Cogn. 2023;49(1):98–115. doi: 10.1037/xlm0001116 35549440

[56] Rayner K. Eye movements in reading and information processing: 20 years of research. Psychological Bulletin. 1998;124:372–422. 9849112 10.1037/0033-2909.124.3.372

[57] Rayner K. Eye movements and attention in reading, scene perception, and visual search. Q J Exp Psychol (Hove). 2009;62(8):1457–506. doi: 10.1080/17470210902816461 19449261

[58] Clifton C, Staub A, Rayner K. Eye movements in reading words and sentences. In: Van Gompel R, Fischer M, Murray W, Hill R, editors. Eye movements: A window on mind and brain. Elsevier. 2007. p. 341–71.

[59] Rayner K, Duffy SA. Lexical complexity and fixation times in reading: effects of word frequency, verb complexity, and lexical ambiguity. Mem Cognit. 1986;14(3):191–201. doi: 10.3758/bf03197692 3736392

[60] Underwood G. Eye guidance in reading and scene perception. Elsevier. 1998.

[61] Liang F, Blythe HI, Bai X, Yan G, Li X, Zang C, et al. The role of character positional frequency on Chinese word learning during natural reading. PLoS One. 2017;12(11):e0187656. doi: 10.1371/journal.pone.0187656 29136002 PMC5685568

[62] Cao HB, Ren L, Han D, Jia DL, Wang JX. The role of morpheme position probability in the recognition of Chinese overlapping ambiguous words. Studies of Psychology and Behavior. 2022;20:732–8.

[63] Lian KY, Ma J, Wei L, Zhang SW, Bai XJ. Developmental study on the role of morpheme position probability clues in Chinese reading aloud. Stud Psychol Behavior. 2021;19:179–85.

[64] Murata M, Ma Q, Uchimoto K, Ozaku H, Utiyama M, Isahara H. Japanese probabilistic information retrieval using location and category information. In: Proceedings of the fifth international workshop on on Information retrieval with Asian languages, 2000. 81–8. doi: 10.1145/355214.355226

[65] Higashiyama S, Utiyama M, Sumita E, Ideuchi M, Oida Y, Sakamoto Y. Proceedings of the 2019 Conference of the North American Chapter of the Association for Computational Linguistics: Human Language Technologies. 2019;2699–709.

[66] Kasisopa B, G Reilly R, Luksaneeyanawin S, Burnham D. Eye movements while reading an unspaced writing system: the case of Thai. Vision Res. 2013;86:71–80. doi: 10.1016/j.visres.2013.04.007 23608059

[67] Wang MG. The inheritance and innovation of traditional Yi calligraphy layout and the correction of a current wrong format. Journal of Honghe University. 2021; 19: 9–12. (in Chinese). doi: 10.13963/j.cnki.hhuxb.2021.03.003

[68] Barnett R, Faggionato C, Meelen M, Yunshaab S, Samdrup T, Hill N. Named Entity Recognition (NER) for Tibetan and Mongolian Newspapers. 2021. doi: arXiv:2104.09804

[69] Wang D, Zeng M, Zhao H, Gao L, Li S, Niu Z, et al. Effects of syllable boundaries in Tibetan reading. Sci Rep. 2023;13(1):314. doi: 10.1038/s41598-022-25759-1 36609398 PMC9822970

[70] Faul F, Erdfelder E, Lang A-G, Buchner A. G*Power 3: a flexible statistical power analysis program for the social, behavioral, and biomedical sciences. Behav Res Methods. 2007;39(2):175–91. doi: 10.3758/bf03193146 17695343

[71] Bates D, Mächler M, Bolker B, Walker S. Fitting linear mixed-effects models Usinglme4. J Stat Soft. 2015;67(1). doi: 10.18637/jss.v067.i01

[72] Liang FF, Wang YS, Zhang MM, Yan GL, Bai XJ. The influence of morpheme familiarity of new words on the promotion of visual word segmentation clues in new word learning. Psychol Sci. 2016;39:258–64. doi: 10.16719/j.cnki.1671-6981.20160201

[73] LIANG F, FENG L, LIU Y, LI X, BAI X. Different roles of initial and final character positional probabilities on incidental word learning during Chinese reading. Acta Psychologica Sinica. 2024;56(3):281. doi: 10.3724/sp.j.1041.2024.00281

[74] Liang F, Ma J, Bai X, Liversedge SP. Initial landing position effects on Chinese word learning in children and adults. J Memory Language. 2021;116:104183. doi: 10.1016/j.jml.2020.104183

[75] Barnett R, Hill N, Diemberger H, Samdrup T. Named-Entity Recognition for Modern Tibetan Newspapers: Tagset, Guidelines and Training Data [Data set]. Zenodo; 2021. doi: 10.5281/zenodo.4536516

[76] Spitzer L, Mueller S. Using a test battery to compare three remote, video-based eye-trackers. In: 2022 Symposium on Eye Tracking Research and Applications. 2022;1–7. doi: 10.1145/3517031.3529644

[77] YAN G, XIONG J, ZANG C, YU L, CUI L, BAI X. Review of Eye-movement Measures in Reading Research. Advances in Psychological Sci. 2013;21(4):589–605. doi: 10.3724/sp.j.1042.2013.00589

[78] Henderson JM, Ferreira F. Effects of foveal processing difficulty on the perceptual span in reading: implications for attention and eye movement control. J Exp Psychol Learn Mem Cogn. 1990;16(3):417–29. doi: 10.1037//0278-7393.16.3.417 2140401

[79] Liversedge SP, Paterson KB, Pickering MJ. Eye Movements and Measures of Reading Time. Eye Guidance in Reading and Scene Perception. Elsevier. 1998. p. 55–75. doi: 10.1016/b978-008043361-5/50004-3

[80] Storn R, Price K. Differential evolution – a simple and efficient heuristic for global optimization over continuous spaces. J Global Optimization. 1997;11(4):341–59. doi: 10.1023/a:1008202821328

[81] Jaeger EL. Negotiating Complexity: A Bioecological Systems Perspective on Literacy Development. Human Development. 2016;59(4):163–87. doi: 10.1159/000448743

[82] You W. Eighth-graders’ reading comprehension of informational texts and literary texts in the 2009 national assessment of educational progress. 2016.

[83] Oh K. Use of reading strategy to assess reading medium effectiveness: Application to determine the effects of reading medium and generation in an active reading task. Virginia Polytechnic Institute and State University. 2013.

[84] Sweller J. Cognitive Load During Problem Solving: Effects on Learning. Cognitive Science. 1988;12(2):257–85. doi: 10.1207/s15516709cog1202_4

[85] Paas F, Tuovinen JE, Tabbers H, Van Gerven PW. Cognitive load measurement as a means to advance cognitive load theory. In: Kalyuga S, editor. Cognitive load theory. Routledge. 2016. p. 63–71.

[86] Rayner K, Fischer MH. Mindless reading revisited: eye movements during reading and scanning are different. Percept Psychophys. 1996;58(5):734–47. doi: 10.3758/bf03213106 8710452

[87] Parker AJ, Slattery TJ. Word frequency, predictability, and return-sweep saccades: Towards the modeling of eye movements during paragraph reading. J Exp Psychol Hum Percept Perform. 2019;45(12):1614–33. doi: 10.1037/xhp0000694 31524433

[88] Demberg V, Keller F. Data from eye-tracking corpora as evidence for theories of syntactic processing complexity. Cognition. 2008;109(2):193–210. doi: 10.1016/j.cognition.2008.07.008 18930455

[89] Payne BR, Stine-Morrow EAL. Aging, parafoveal preview, and semantic integration in sentence processing: testing the cognitive workload of wrap-up. Psychol Aging. 2012;27(3):638–49. doi: 10.1037/a0026540 22229390 PMC3376187

[90] Li X, Pollatsek A. An integrated model of word processing and eye-movement control during Chinese reading. Psychol Rev. 2020;127(6):1139–62. doi: 10.1037/rev0000248 32673033

[91] Liang FF, Xiang Y, Long ML. Initial morpheme position probability information does not participate in word segmentation in Chinese reading: evidence based on parafoveal processing. Stud Psychol Behavior. 2022;20:318–25.

